# Assessing the adversarial robustness of multimodal medical AI systems: insights into vulnerabilities and modality interactions

**DOI:** 10.3389/fmed.2025.1606238

**Published:** 2025-07-24

**Authors:** Ekaterina Mozhegova, Asad Masood Khattak, Adil Khan, Roman Garaev, Bader Rasheed, Muhammad Shahid Anwar

**Affiliations:** ^1^Machine Learning and Knowledge Representation Laboratory, Innopolis University, Innopolis, Russia; ^2^Department of Computing and Applied Technology, College of Technological Innovation, Zayed University, Abu Dhabi, United Arab Emirates; ^3^School of Computer Science, Hull University, Hull, United Kingdom; ^4^Laboratory of Innovative Technologies for Processing Video Content, Innopolis University, Innopolis, Russia; ^5^IRC for Finance and Digital Economy, King Fahd University of Petroleum and Minerals, Dhahran, Saudi Arabia

**Keywords:** machine learning (ML), adversarial attack, multimodal data fusion, classification, X-ray

## Abstract

The emergence of both task-specific single-modality models and general-purpose multimodal large models presents new opportunities, but also introduces challenges, particularly regarding adversarial attacks. In high-stakes domains like healthcare, these attacks can severely undermine model reliability and their applicability in real-world scenarios, highlighting the critical need for research focused on adversarial robustness. This study investigates the behavior of multimodal models under various adversarial attack scenarios. We conducted experiments involving two modalities: images and texts. Our findings indicate that multimodal models exhibit enhanced resilience against adversarial attacks compared to their single-modality counterparts. This supports our hypothesis that the integration of multiple modalities contributes positively to the robustness of deep learning systems. The results of this research advance understanding in the fields of multimodality and adversarial robustness and suggest new avenues for future studies focused on optimizing data flow within multimodal systems.

## 1 Introduction

Deep learning systems have demonstrated rapid development and are currently being extensively applied in a wide range of fields, including healthcare. The medical domain is especially promising for AI integration due to the variety of existing tasks that involve diverse data types, such as texts, images, and numerical recordings (1). Common examples of medical data include X-ray images, CT scans, and MRIs images representations, Electronic Health Record (EHR), text prescriptions, and more (2, 3). Task-specific models are commonly used to analyze these data types for applications such as disease prediction, anomaly detection, vaccine design, drug discovery, and more (4). Along with single-modality models, general-purpose multimodal large models have recently emerged, offering the potential to process these different data simultaneously and address even more complex tasks (1).

Although the healthcare domain presents significant opportunities for AI innovation, it also imposes high standards on these systems, requiring exceptional performance, reliability, robustness, and interpretability. This raises critical questions about the vulnerabilities of these systems. Specifically, deep learning models frequently remain vulnerable to adversarial attacks—small, often imperceptible, perturbations to the input data, capable of misleading model predictions (5). Studies have shown that medical AI models can be highly vulnerable to adversarial attacks (6–9). Due to the healthcare realm being an area with high demands to systems accuracy and robustness, it is important to thoroughly understand the vulnerabilities of these models to ensure their reliability and safety in medical applications.

In this research, we take a step forward in the exploration of a new and relatively unexamined topic: adversarial attacks across modalities, with the aim of uncovering new patterns in the robustness of multimodal models. We successfully deceived AI models specialized in medical tasks by employing adversarial attacks on two modalities: images and texts. We observed that the models are indeed vulnerable to these attacks, with varying levels of damage depending on the severity of the attack.

Through our further experiments, we demonstrate that multimodality can improve the overall performance of the model. Additionally, combining modalities can also result in enhanced robustness of the model. In our experiments, we applied adversarial attacks on different data types; however, the multimodality models appeared to be more robust to these attacks compared to single-modality models.

We suggest that further research into how data flows in multimodal AI models might be a key to studying the robustness of multimodal AI systems.

This paper is structured as follows. Section 2 examines the vulnerabilities of both general and medical AI systems toward adversarial attacks and reviews similar approaches to enhancing their robustness. Section 3 outlines the methodology established for conducting our experiments, with the detailed description and obtained results discussed in Section 4. Section 5 discusses the findings, shares key insights, and Section 6 concludes the paper with a brief research summary and potential future directions.

## 2 Literature review

We conducted a literature review to examine the current state of AI systems in the healthcare domain and their practical implementations in this field. Currently, some task-specific models are already being employed for applications such as disease prediction, anomaly detection, vaccine design, drug discovery, and more. For instance, Electronic Health Records (EHR) are frequently used for anomaly detection and risk assessment, medical imaging modalities, such as X-rays, CT scans, and MRIs are used for disease prediction (2–4). Other prominent examples of successful implementations of AI models in healthcare include CheXNet, a convolutional neural network (CNN) for pneumonia prediction based on chest X-ray images; diagnosis prediction systems using EHR; MURA for bones abnormality detection, and ToxDL, a CNN-based model for assessing protein toxicity (2, 10, 11).

Our review also explored adversarial vulnerabilities in ML models. Research demonstrated that adversarial attacks have already been extensively studied, and it has been proven that both models with known and unknown internal parameters can be attacked. These attacks can deceive the model, forcing it to generate incorrect results—either randomly (untargeted attacks) or specifically (targeted attacks). Goodfellow demonstrated that adversarial attacks can compromise a wide range of models: not only deep learning models but also linear models, such as softmax regression (5). Furthermore, these attacks can target various data modalities.

Regarding the text modality, attacks applied on texts are designed to alter different textual units: characters, words, or phrases. The most common text attacks include word flipping, word swaps, word deletions or additions (12), and synonym replacements (13). These techniques can rely on methods such as word embeddings or contextual language models such as BERT to choose replacements that preserve meaning (14).

In the context of images, attacks on visuals primarily involve gradient-based methods, with the most popular being FGSM (Fast Gradient Sign Method) (5) and PGD (Projected Gradient Descent) (15). These attacks perturb the input data in the direction of the gradient of the model's loss function with respect to the input, aiming to mislead the model.

Studies have shown that medical AI models can be highly vulnerable to adversarial attacks due to several reasons, including complexity of medical images, overparameterization of medical AI models (6, 7). Another factor is that they are frequently based on pre-trained architectures, and information about the model can provide attackers with a significant advantage, enabling them to manipulate the input to exploit the model's vulnerabilities. Additionally, if the data types remain consistent, attackers can target specific input patterns that the model expects, making it easier for them to craft adversarial examples (6, 7).

The study of robustness of multimodal models is a relatively new and developing field, with a few research experimenting with attacks on these models. Some studies propose ideas that multimodaliity can improve robustness (16). However, other research has experimentally shown that random fusion techniques do not provide advantages for model robustness (16, 17), while others suggest that improvements are possible only with specifically crafted fusion techniques (16). Huang et al. (18) try to close this gap by developing the adversarial attack called *2M-attack* on medical multimodal models. Thota et al. (19) use the modification of PGD attack to compromise the Language-Image model and show that such model is vulnerable against even small adversarial perturbations. In our study, we would like to investigate the impact of various fusion techniques on the total model robustness.

## 3 Method

### 3.1 Framework concept

In this section, we introduce the general concept of our methodology and present an overview of our experimental setup.

This study focuses mainly on two modalities—images and text—since they are the most commonly encountered in healthcare applications (20).

We initially constructed two separate models: an image-based model *M*_*I*_ and a text-based model *M*_*T*_. We then combined *M*_*I*_ and *M*_*T*_ to create a multimodal model, *M*_*IT*_, resulting in three distinct models.

We apply different attack scenarios on these models and evaluate the models' robustness against these attacks. First, we implement Fast Gradient Sign Method (FGSM) and Projected Gradient Decent (PGD) attacks on the visual model. PGD attack can be considered as We apply attacks on the language model, which include synonym substitution, denoted as “*Synonym replacing*,” and words deletion, denoted as “*Half-sentence deleting*.” For the multimodal model *M*_*IT*_, we test each of the mentioned attacks individually. For example, if we attack *M*_*I*_ part of the model, text description remain unchanged. Finally, we combine text and image attacks to challenge both modalities.

The goal is to investigate how the attack of one modality influences the overall performance of the multimodal model. Afterward, we apply attacks on the second modality to observe how the model's performance degrades. This approach should help to test the hypothesis regarding the dominance of modalities in enhancing multimodal models' adversarial robustness. Another hypothesis we aim to test is whether multimodal models are inherently more robust to adversarial attacks due to their multimodal nature.

In the following section, we elaborate on the technical details related to the implementation of the proposed experiment.

### 3.2 Models

#### 3.2.1 CNN

For handling image data, we used a pre-trained SE-ResNet-154 model. Pre-trained architectures, such as ResNet50 (10) and SE-ResNet-154 (21), have demonstrated effectiveness in solving medical imaging tasks, such as chest X-ray classification. For instance, Rajpurkar et al. in their study (10) used ResNet-50, while we utilized a more advanced model, SE-ResNet-154, which incorporates a squeeze-and-excitation block and is expected to provide improved performance over ResNet-50 for this task. Thus, for this research, we used SE-ResNet-154 as the base model and fine-tuned it by adding a custom classification layer. We utilized this model for the binary classification task for predicting whether a person's X-ray image is normal or has any anomalies.

#### 3.2.2 Language model

For handling the text modality, we utilized the pre-trained Bio_ClinicalBERT model. This model is based on BioBERT (22), a state-of-the-art architecture, and is trained on the large MIMIC-III dataset containing electronic health records (23).

BioBERT is considered as one of the best medical models and MIMIC_III is one of the top datasets.

For this study, we fine-tuned Bio_ClinicalBERT specifically for clinical text accompanying medical images, making it well-suited for our task. This model solved the same binary classification task as *M*_*I*_ but with the text labels as inputs.

#### 3.2.3 Modality fusion

To build an effective multimodal model, it is crucial to understand the methods for combining different modalities. The main approaches include early fusion (also known as feature-level fusion), late fusion (decision-level fusion), and attention-based techniques. Among these, early and late fusion are two fundamental paradigms in multimodal integration, and thus, they are the primary focus of this study.

Early fusion is generally considered the best option when model parameters are known and the dataset is large since it allows for a unified representation of modalities at the feature level, leveraging the full richness of the combined data (22).

However, in practical scenarios where dataset sizes are moderate, late fusion often proves to be more effective. By treating each modality independently and combining their decision-level outputs, late fusion can better utilize the available samples to make accurate predictions, especially when the separability of individual modalities is comparable (22). Thus, we used both fusion techniques. Accordingly, we implemented two models for classification: VisionBERT_EarlyFusion and VisionBERT_LateFusion. The multimodal model aimed to predict whether a person has a disease or is healthy based on chest X-ray images accompanied by text labels.

##### 3.2.3.1 VisionBERT_EarlyFusion

**Figure 1:**
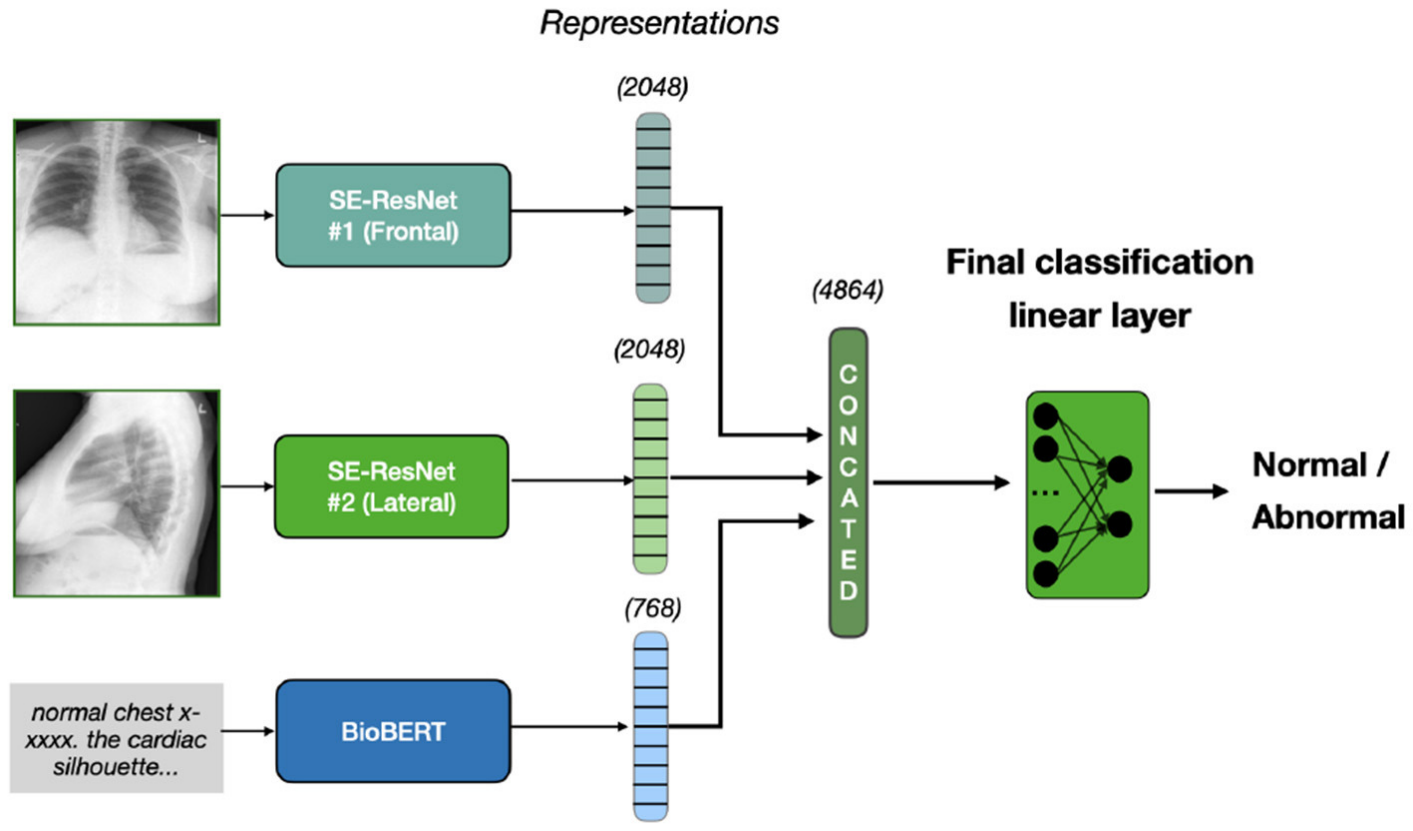
Early fusion approach. Two X-rays, frontal and lateral, are inputted into SE-ResNet models, producing image features of 2048 dimensions each. Text diagnosis is processed by BioBERT, producing a 768-dimension representation. These are concatenated to form a 4864-dimension vector, which a linear layer classifies as normal or abnormal.

This model combines lateral and frontal images using the SE-ResNet-154 architecture for feature extraction, excluding the final fully connected layer to obtain spatial features. These image features are concatenated and fused with the textual features from BERT's [CLS] token representation. The fused features are passed through a linear layer for binary classification (normal/abnormal). We take the pre-trained weights and train all three extracion models and classification head simultaneously on our dataset. This approach is illustrated on Figure 1.

##### 3.2.3.2 VisionBERT_LateFusion

**Figure 2:**
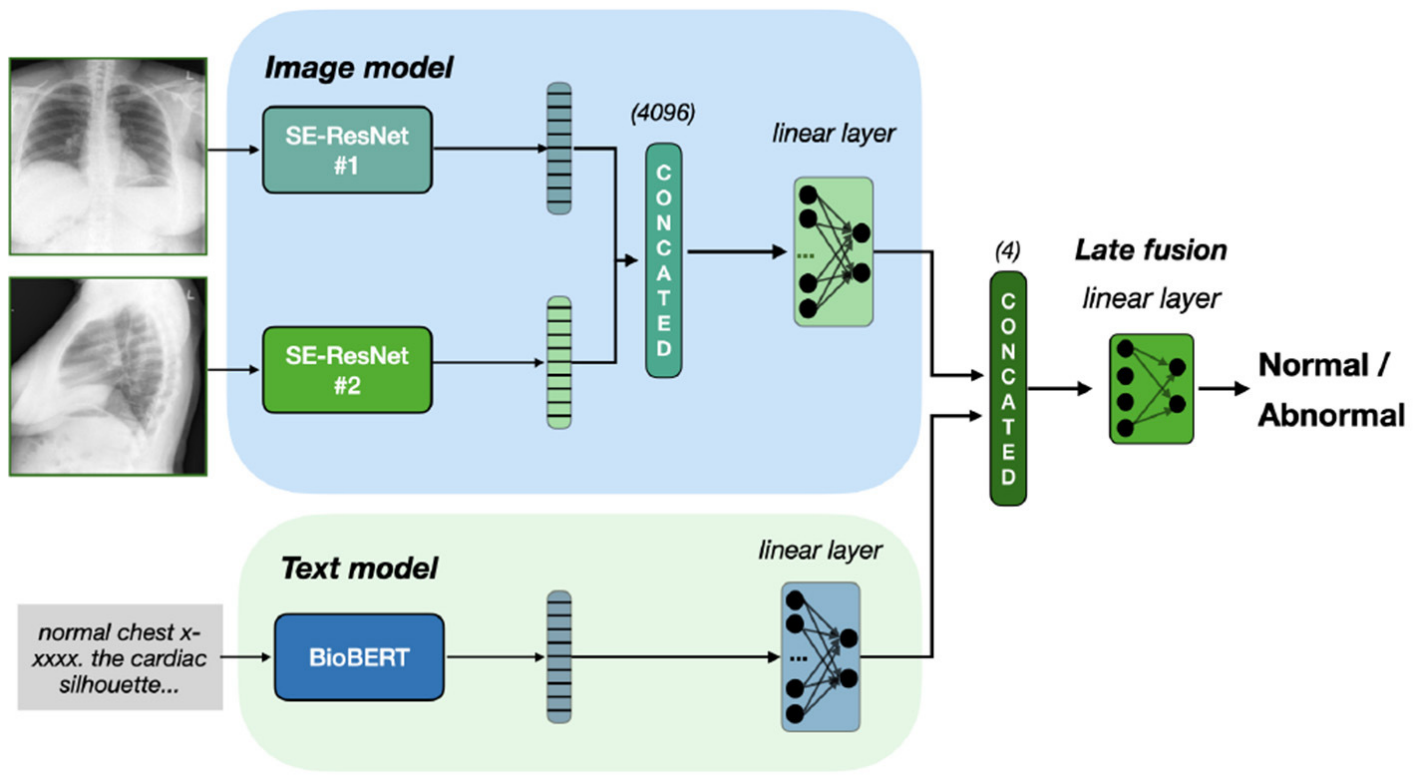
Late fusion of Se-ResNet-s and BioBERT. We train separately image and text models on classification task. To fuse the final prediction, we freeze the models weights and train the linear layer on concatenated prediction.

Similar to the VisionBERT_EarlyFusion model, this architecture extracts features from both the image (via SE-ResNet-154) and text (via Bio_ClinicalBERT). However, late fusion is applied: separate classifiers for each modality produce independent predictions, which are concatenated and passed to a final classifier for decision-making. This enables the model to learn the contributions of each modality before fusion. Thus, the training contains of two stages. On the first stage, we train image and text classifiers separately. On the second stage, we freeze their weights and train the final classification layer, with four input and two output neurons. Our late fusion model is presented on Figure 2.

**Figure 3:**
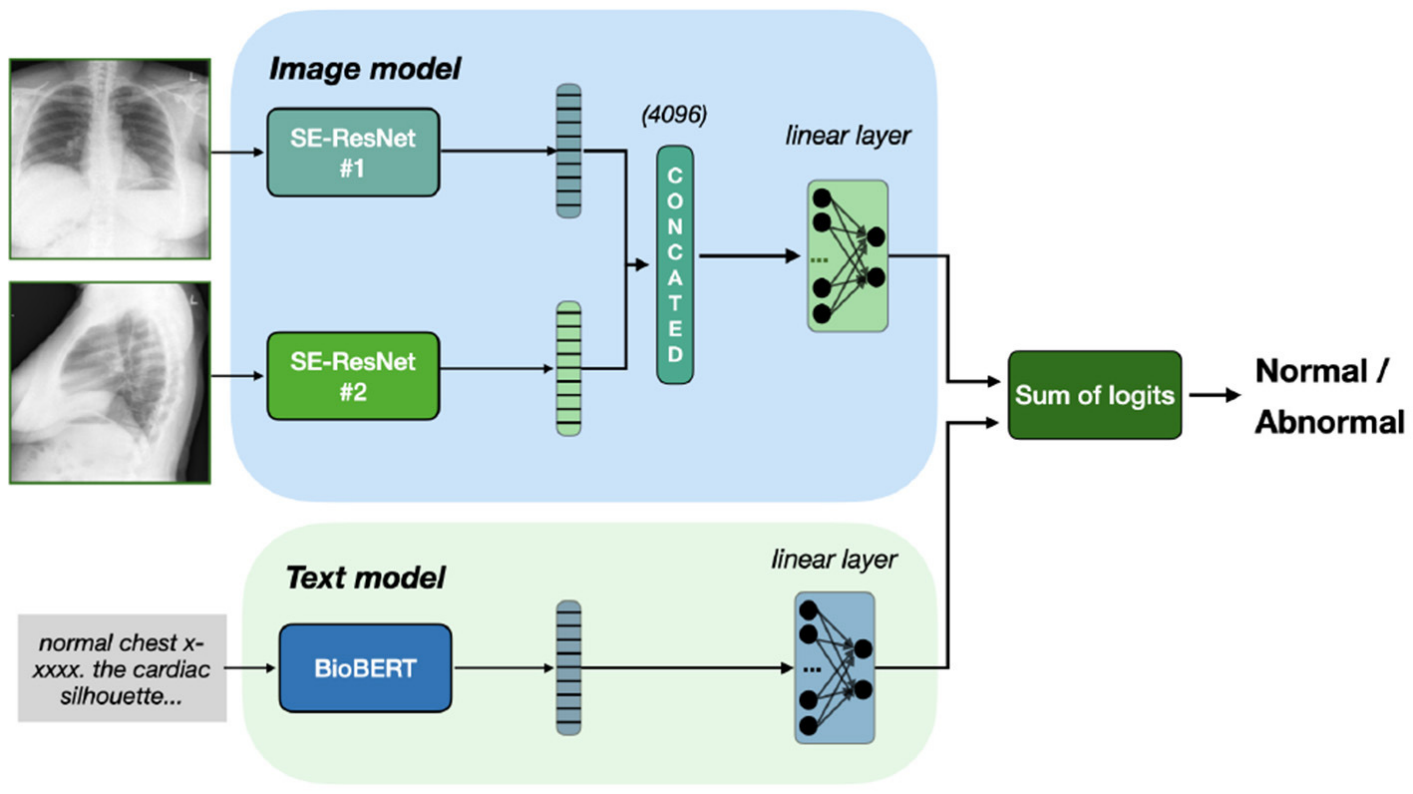
Ensemble fusion of Se-ResNet-s and BioBERT. Outputs from both models sum up, resulting in classification based on the sum of logits, with no additional training of fusion head.

Additionally, on Figure 3 we present a special case of late fusion called *ensemble fusion*, where we do not train the final classifier layer and just consider the sum on predictions from image and text models. In comparison to late fusion, the ensemble fusion is simpler and threat two modalities equally.

### 3.3 Dataset

We used a multimodal dataset collected by Indiana University that incorporates chest X-ray images accompanied by text captions. This dataset consists of two parts:

- **indiana_reports.csv**
  This file includes the following columns:

- uid
- MeSH
- Problems
- image
- indication
- comparison
- findings
- impression
- Label

- **indiana_projections.csv**
  This file includes the following columns:

- uid
- filename
- projection (either “frontal” or “lateral”)

The data consists of 3,999 entries, corresponding to the number of image pairs (lateral and frontal images) and associated textual notes. Approximately 36% of the entries are labeled as normal, with other entries having signs of disease.

We combined information from indiana_reports.csv and indiana_projections.csv to create the following multimodal dataset:

- uid
- frontal_image
- lateral_image
- text_caption
- diagnosis

**Figure 4:**
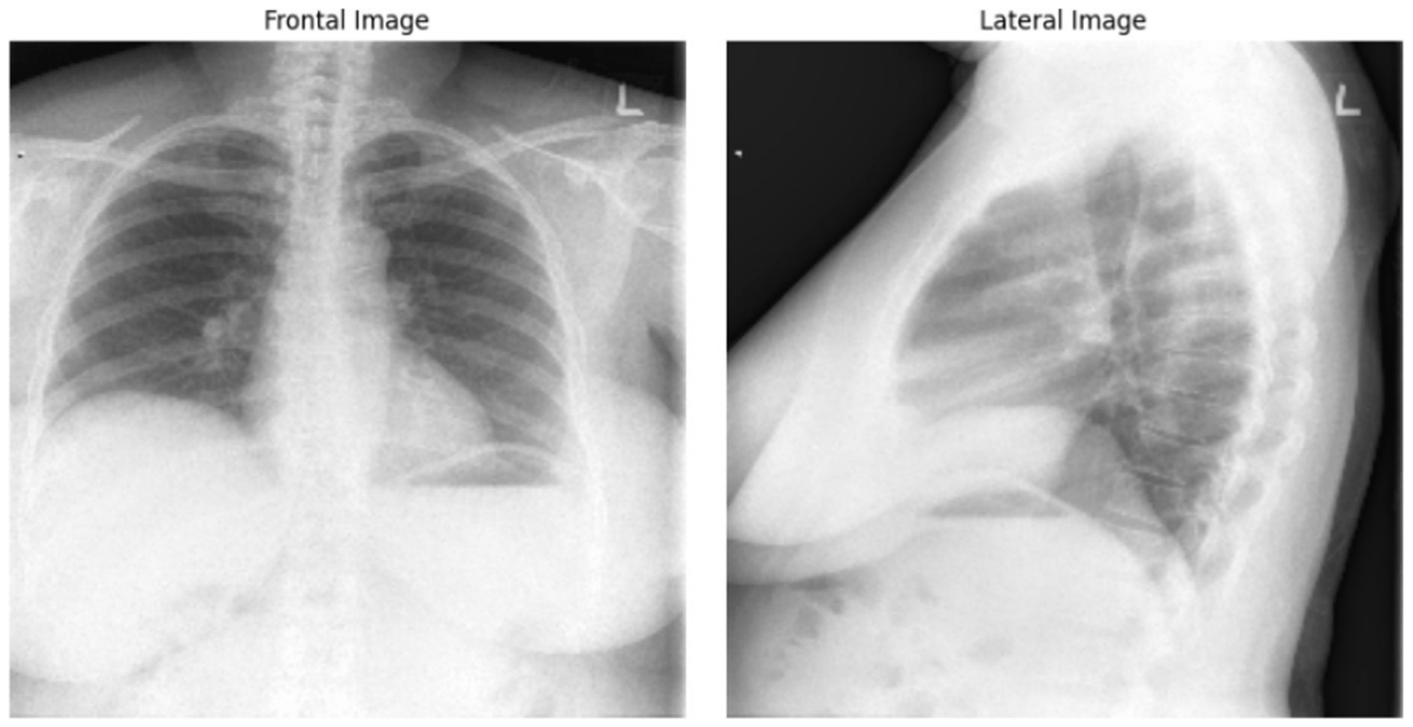
Frontal and lateral view of Chest X-ray images. The example from “Chest X-rays” dataset of Indiana University.

Example of Chest X-ray images from the dataset is presented on Figure 4.

To retrieve the text description, we combined the Impression, Findings, and Indication columns. We used both the frontal and lateral chest X-ray images from this dataset as the input for the vision model *M*_*I*_.

### 3.4 Attack configurations

We aimed to implement attacks on two modalities in this study: text and images. In our research, we implemented word deletion and synonym substitution attacks with varying levels of intensity, tuning them by adjusting the percentage of textual units we perturb. We chose these attacks because they are among the most common approaches, straightforward, and effective (12–14). Specifically, we tested half-word deletion, where 50% of the words are removed. Another text attack, synonym substitution, involved replacing a fraction of the words in the text caption with their synonyms. We tested substitution fractions of 20% and 40%.

On the images, we implemented the FGSM and PGD attacks, as they are the most common approaches, and tuned the hyperparameter ϵ to define the intensity of the attack. Specifically, we used $\epsilon =\frac{8}{255}$, as the most common choice in the literature (5, 15), and ϵ = 0.2, as the extreme aggressive perturbation.

### 3.5 Training and validation setup

During the data preprocessing phase, we initially divided the permuted dataset into training and testing subsets in an 80% to 20% ratio, respectively. Subsequently, all models were trained using the same portion of the dataset to ensure consistency. To facilitate a fair comparison among the models, we minimized unnecessary transformations during both the training and evaluation phases. For the lateral and frontal images, we applied normalization using a mean of 0.61 and a standard deviation of 0.24, calculated from the training dataset. Additionally, the text descriptions were converted to lowercase and stripped of extraneous whitespace. We evaluated the models using accuracy and F1-score as the main metrics since the dataset is not balanced.

## 4 Experiments

### 4.1 Framework implementation

#### 4.1.1 CNN

The vision model *M*_*I*_ is built using transfer learning with a pre-trained SE-ResNet-154 architecture. We added a custom classification layer to the model for task-specific fine-tuning. The classifier layer is designed to handle the concatenated feature maps from the SE-ResNet-154 output.

For training, we used the following hyperparameters:

- Batch size: 128
- Epochs: 13
- Optimizer: Adam
- Learning Rate: 1e-4
- Scheduler: ReduceLROnPlateau

#### 4.1.2 Language model

We post-trained the Bio_ClinicalBERT model for 5 epochs using Adam with a learning rate of 2 × 10^−5^, which is commonly used for fine-tuning transformer models. The Binary CrossEntropyLoss function is applied for the loss calculation.

#### 4.1.3 VisionBERT_EarlyFusion

**Training Parameters**:

- Optimizer: Adam
- Learning Rate: 1 × 10^−4^
- Epochs: 5

#### 4.1.4 VisionBERT_LateFusion

**Training Parameters**:

- Optimizer: Adam
- Learning Rate: 1 × 10^−5^
- Epochs: 5

## 5 Results

### 5.1 Key findings

**Figure 5:**
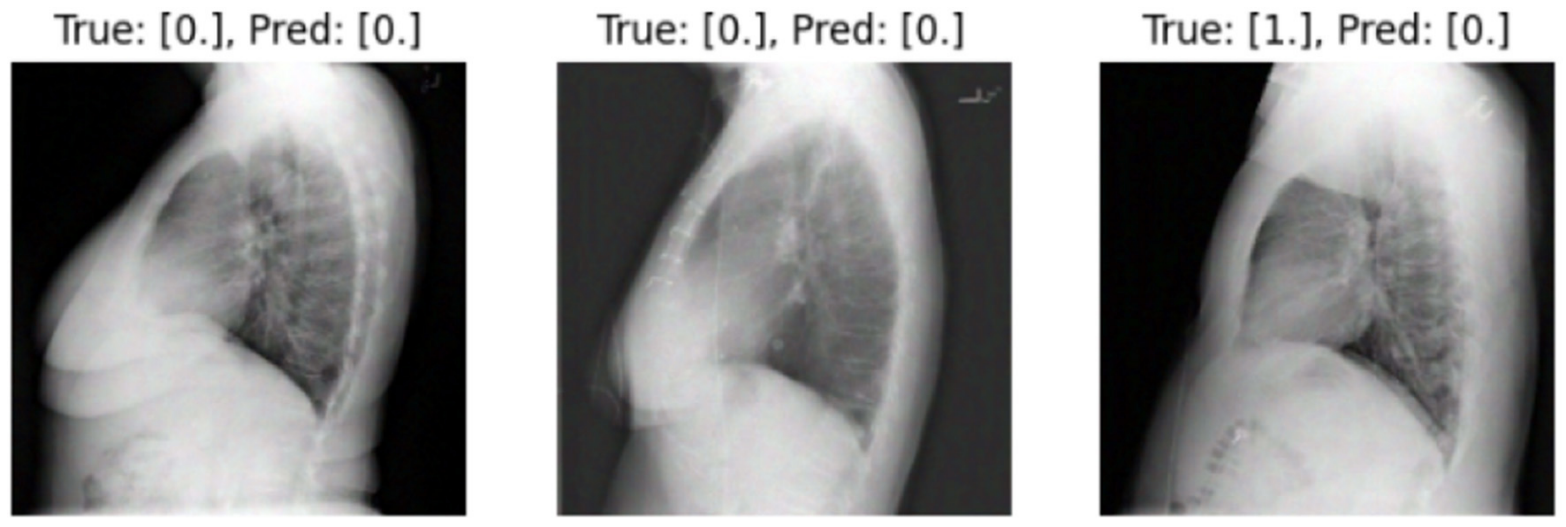
FGSM attack with ϵ = 0.03. Predicted labels (named “Pred.”) are gathered from VisionBERT_EarlyFusion model.

**Figure 6:**
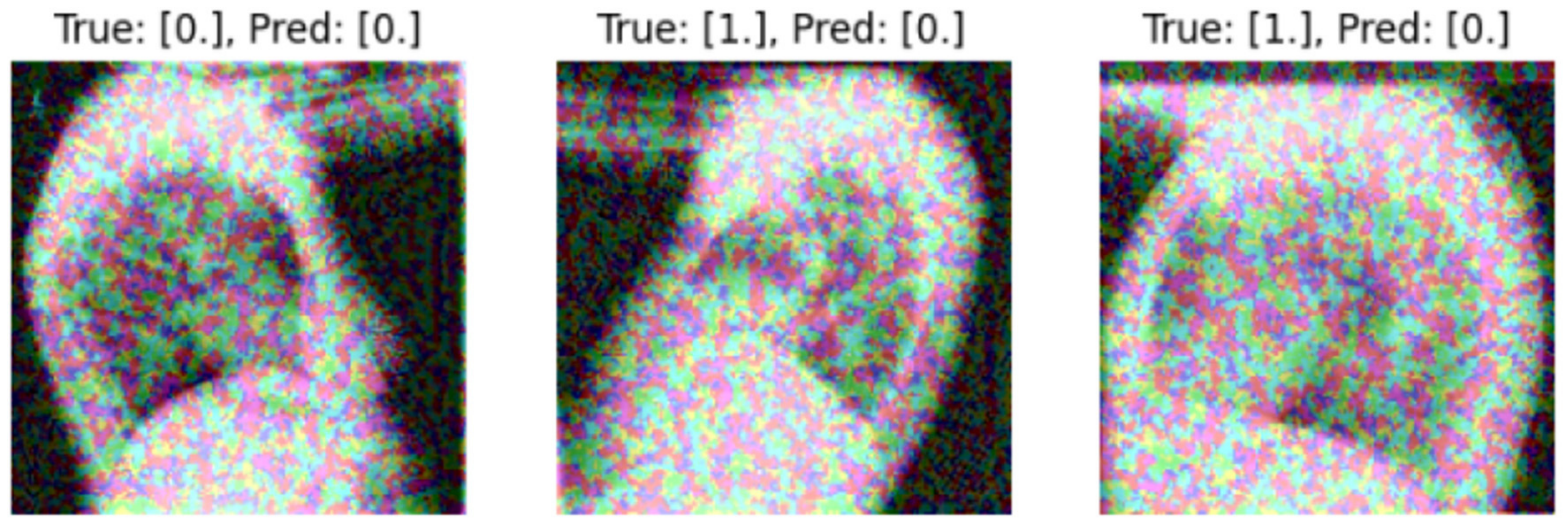
FGSM attack with ϵ = 0.2.Predicted labels (named “Pred.”) are gathered from VisionBERT_EarlyFusion model.

We present some examples of the adversarially generated images from the multimodal dataset under FGSM attack on Figures 5, 6. As seen in the images, adversarial attacks with quite moderate parameters result in images, which look imperceptibly different from the original images, and the model *M*_*IT*_ maintains high accuracy. However, the accuracy of *M*_*IT*_ degrades significantly under the attacks with high perturbation budget for ensemble and early fusion models.

In the following boxes we show the successful examples of “Synonym replacing” attack, which is heavily based on WordSwapWordNet^1^ attack from textattack package (24).

**Table:** 

**Example 1:**
**Impression:** No acute pulmonary disease.
**Findings:** The lungs are brighten. There is no pleural effusion or pneumothorax. The heart and mediastinum are normal. The skeletal structures are normal.
**Indication:**
**Chest pain**
**Label:** Abnormal

**Table:** 

**Example 2:**
**Impression:** cold-shoulder megacardia. Clear lungs. No effusion
**Findings:** nan
**Indication:** chest pain dyspnea
**Label:** Normal

**Table:** 

**Example 3:**
**Impression:** No acute cardiopulmonary disease
**Findings:** The lungs are authorize. The heart and pulmonary XXXX appear normal. Pleural infinite are unmortgaged. The mediastinal contours are convention. Cadaverous overlap in the lung apices could unsung a small pulmonary nodule.
**Indication:** V70.0 ROUTINE XXXX MEDICAL EXAMINATION AT A XXXX XXXX FACILITY 305.1 NONDEPENDENT TOBACCO APPLY XXXX
**Label:** Normal

In Table 1, we present f1-scores for early, late and ensemble fusions of our VisionBERT model. To test them, we apply various adversarial attacks both separately on image and text modalities and the their combination. In general, the late fusion approach employed by our VisionBERT model exhibits superior performance compared to other models, despite the individual modalities being susceptible to corresponding adversarial attacks (refer to the figures in brackets in Table 1). Conversely, the ensemble fusion method, which represents the simplest integration of image and text models, demonstrates the lowest resilience against such attacks. This discrepancy in performance may be attributed to the nature of late fusion, which generates a weighted combination of predictions from both image and text modalities.

**Table 1:** F1-score of models under different attack types.

**Attack type**	**VisionBERT_EarlyFusion**	**VisionBERT_LateFusion**	**VisionBERT_EnsembleFusion**
No attack	94.94	93.73	91.88
FGSM, ϵ = 0.03	93.65	93.32 *(49.28)*	84.45
FGSM, ϵ = 0.2	83.48	79.05 *(0.0)*	48
PGD, ϵ = 0.03, steps = 10	90.54	92.25 *(0.0)*	14.65
PGD, ϵ = 0.2, steps = 10	18.67	83.51 *(0.0)*	3.97
Synonym replacing	49.6	33.04 *(37.32)*	57.22
Half-sentence deleting	79.94	79.68 *(81.08)*	80.66
FGSM(ϵ = 0.03) + Synonym replacing	31.10	42.78	29.81
PGD(ϵ = 0.03) + Synonym replacing	**12.54**	**31.34**	**0.7**
FGSM(ϵ = 0.03) + Half-sentence deleting	58.16	55.16	53.88
PGD(ϵ = 0.03) + Half-sentence deleting	46.56	48.05	9.86

First four attack are related to image attacks, next two attacks targets the text modality, and the rest are combination of the previous attacks. F1-score in the brackets for VisionBERT_LateFusion model stands for the performance of the single modality.

We also analyze the transferability of adversarial examples between our models. The transferability is the important feature of adversarial examples which allows to attack one model and successfully use the resulting perturbed data on another model. Such scenario is called “black-box”, because the adversary may not seen the target model and attack the substitute model. We report the results of PGD attacks transferring with $\epsilon =\frac{8}{255}$ and ϵ = 0.2 in Tables 2, 3, respectively. The experiment demonstrates that the adversarial images for the late and early fusion models do not transfer well, as we don't see the same drop of accuracy as in Table 1. Note that in all cases the text model is not attacked.

**Table 2:** Transferability of PGD-attacked ($\epsilon =\frac{8}{255}$) images between the models.

**Black-box/Generator**	**VisionBERT_EarlyFusion**	**VisionBERT_LateFusion**	**VisionBERT_EnsembleFusion**
VisionBERT_EarlyFusion	-	94.35	93.93
VisionBERT_LateFusion	93.96	-	92.25
VisionBERT_EnsembleFusion	93.86	94.86	-

“Generator” models are used to create the adversarial images which are fed to the corresponding “Black-box” models.

**Table 3:** Transferability of PGD-attacked (ϵ = 0.2) images between the models.

**Black-box/Generator**	**VisionBERT_EarlyFusion**	**VisionBERT_LateFusion**	**VisionBERT_EnsembleFusion**
VisionBERT_EarlyFusion	-	94.37	94.55
VisionBERT_LateFusion	93.57	-	82.78
VisionBERT_EnsembleFusion	93.86	0	-

### 5.2 Discussion

As shown in the experiments, both single-modality models and multimodal models are vulnerable to adversarial attacks, though with different intensities. While even gentle attacks with small parameters significantly degraded the performance of single-modality models, the multimodal model only experienced significant accuracy drop under exceptionally strong attacks.

Another point we want to mention concerns the multimodality domain. Although our vision model alone exhibited poor performance, VisionBERT benefited from the strong performance of the effective language model, which contributed to its overall success.

The multimodal model VisionBERT demonstrated exceptional performance and relative robustness against various types of attacks on different modalities. Although attacks reduced the model's accuracy, it still outperformed single-modality models under similar conditions. So, multimodality can not only enhance the overall performance by combining the strengths of the individual models it integrates, but it can also increase the overall robustness to adversarial scenarios.

## 6 Conclusion

Studying the robustness of AI models in the healthcare domain is essential. Special focus should be given to multimodal models, which are widely used in various tasks due to their versatility and potential to enhance adversarial robustness. In our study, we observed interesting behavior in multimodal models and examined their resilience under different adversarial scenarios. For this research, we implemented two single-modality models: SE-ResNet-154 model for prediction whether a person has some medical issues or not based on chest X-ray images, and a BioBERT-based language model for the same binary classification task with the text labels for the same patients as inputs. Subsequently, we created a multimodal model by integrating these two single-modality models.

Our experiments demonstrate that all models can be attacked by adversarial examples, but the multimodal model appears to be more resilient to such perturbations. We attribute this behavior to the multimodal nature of the model. We propose that further research is needed in both the domain of multimodality AI models and adversarial attacks on such models. Understanding how information flows across modalities is particularly intriguing. This insight could enhance our understanding of how deep learning models work, which makes this study particularly significant.

In our future work, we would like to put more attention should be given to the fusion techniques for combining modalities since it can also significantly influence the results.

## Data Availability

Publicly available datasets were analyzed in this study. This data can be found at: Chest X-rays (Indiana University) (https://www.kaggle.com/datasets/raddar/chest-xrays-indiana-university).

## Footnotes

^1^Documentation of the attack.
